# Early warning and advisory can contribute to enhanced wheat stem and yellow rust management in Ethiopia

**DOI:** 10.1038/s41598-026-67016-9

**Published:** 2026-08-14

**Authors:** Michael Euler, Gerald Blasch, Yoseph Alemayehu, Ayele Badebo, Jacob W. Smith, Tamás Mona, Christopher A. Gilligan, David P. Hodson

**Affiliations:** 1 International Maize and Wheat improvement Center (CIMMYT), ILRI Sholla Campus, Addis Ababa, Ethiopia; 2 Present Address: CIMMYT, Carretera México-Veracruz Km. 45, El Batán, Texcoco, México; 3 Department of Plant Sciences, University of Cambridge, Downing Street, Cambridge, UK; 4 CIMMYT, Lalitpur, Nepal

**Keywords:** Wheat rust, Disease early warning and advisory, Farmer fungicide use, Ethiopia, Environmental sciences, Plant sciences

## Abstract

Wheat rusts are major constraints for global wheat production, leading to substantial yield and economic losses. To mitigate rust related production risks, disease forecast models generate time and location-specific information on the emergence and spread of wheat rusts. While predictions become increasingly more accurate, information on whether model outputs can enhance the effectiveness of farmers’ disease management is scarce. In this paper, we used farm household survey data and duration analysis to assess the responsiveness of smallholder farmers to risk predictions for wheat yellow and stem rust. The study used: (i) rust pressure forecasts from the wheat rust Early Warning and Advisory System (EWAS) that was first piloted in 2015 and routinely deployed in Ethiopia since 2017; (ii) within-season data on fungicide applications from 520 farmers from Ethiopia’s major wheat production regions. Results from duration models indicated that the timing of farmers’ fungicide applications for wheat rust control was associated with increasing predicted rust pressure levels and farmer exposure to related extension services. However, farm-level fungicide application dates showed that about one fourth of all applications occurred outside critical growth stages of wheat. We discuss how the existing operational rust surveillance system can enhance farmers’ wheat rust management.

**Supplementary Information:**

The online version contains supplementary material available at https://doi.org/10.1038/s41598-026-67016-9.

## Introduction

Wheat rusts are major biological constraints for global wheat production and food supply, yet estimates of annual yield and economic losses are scarce^1^. Global losses from wheat yellow, leaf and stem rusts were estimated at 20–30 million metric tons annually, corresponding to US$ 3.6–5.4 billion per year^2–4^. Ethiopia is the largest wheat producer in sub-Saharan Africa and a known hotspot for diseases. In 2023, wheat was grown on around 1.9 million hectares with a total production of 5.8 million metric tons^5^. Further area increases are anticipated with the widespread cultivation of irrigated wheat in a quest for self-sufficiency^6^. Although average yield levels have grown from 1.8 metric tons in 2010 to 3.1 metric tons per hectare in 2023^5^, recurrent rust outbreaks continue to challenge sustained growth in wheat production with yellow rust concentrated in the south-western and western districts, stem rust in the southern and south-eastern districts, and leaf rust in the central-southern and parts of northern Ethiopia^7^. Rust-related economic losses have been valued at tens of millions US$ per year^7,8^. Ethiopia is particularly vulnerable to wheat rust, for instance, two of the major rust incidences in the recent past include the yellow rust *(Puccinia striiformis* f. sp. *tritici)* epidemic of 2010 which affected over 38% of Ethiopia’s wheat-growing area^8^ and resulted in national production loss of at least 15%^9^, and the stem rust *(Puccinia graminis* f. sp. *tritici)* epidemic of 2013-14 which caused yield losses of up to 92% for the susceptible and widely grown wheat cultivar Digalu in the worst-affected areas^9^.

In response to these epidemics, in 2017, a wheat rust Early Warning and Advisory System (EWAS) was deployed, following two years of pilot introduction and testing^10^. Within the EWAS, the dispersal of infectious urediniospores (hereafter ‘spores’) and environmental suitability for infection at spore deposition sites are modelled based upon meteorological forecasts and near-real time field surveys for wheat rust. The EWAS provides an automated 7-day forecast at the district (*Woreda*) level on the risk of rust outbreaks for all wheat-growing areas in the country. This spatial forecasting is particularly relevant given the strong heterogeneity in rust prevalence across agroecological zones. Yellow rust prevalence generally increases with altitudes, while stem rust prevalence tends to decrease^7^. However, these general associations between altitude and rust prevalence vary across locations and seasons, as rust occurrence is also shaped by other environmental, agronomic, and epidemiological factors. Rust pressure forecasts and wheat rust field surveys inform national planning for fungicide procurement and distribution across the country. Information on rust pressure levels is also disseminated to farmers through a network of national agricultural extension agents, with the objective of enhancing timely and informed management decisions. At the farm level, the most effective rust mitigation strategy is the planting of wheat varieties with rust resistance traits^12^. Although smallholders have widely adopted rust-resistant varieties across Ethiopia’s major wheat-growing areas^13^, no variety offers long term (durable/race-nonspecific) protection against yield losses, as resistance based on single race-specific genes can be overcome by newly emerging fungal races in any season^7,14^. In the absence of varietal resistance, strategies to minimize rust-related yield losses include the application of fungicides within a growing season^12^.

While the use of rust-resistant cultivars has been documented^12^, there is little empirical evidence on farmers’ fungicide use practices in response to wheat rust pressure and farmer exposure to related early warning and advisory services. This is a critical gap since chemical control measures are only effective in minimizing the incidence and severity of wheat rust when fungicide applications fall within critical growth stages of wheat, specifically when the plant is in a rust-susceptible growth stage and when there is high rust pressure. In these conditions, non-application would likely result in infection and yield loss. Moreover, optimized timing and dosage of fungicide application can lead to increases in farm revenues^15^, reduce negative externalities for human and environmental health^16,17^, and delay the build-up of fungicide-resistant rust strains^18^.

In this paper, we used farm household survey data to assess the responsiveness of smallholder farmers to EWAS risk predictions for wheat rusts. Previous research has shown that farmers’ input use decisions can be enhanced by providing farm-specific information and advisory services^19,20^. For instance, in Ecuador, farmer use of integrated pest management practices in potato production was enhanced after their exposure to repeated within-season extension information and advisory text-messages^21^. Also in Ecuador, recurring within-season messaging led to higher adoption of recommended management practices among blackberry farmers^22^. In the case of wheat rust, we expect farmer exposure to location-specific rust pressure predictions to enhance the effectiveness of fungicide applications throughout the season. Our study aims at contributing to a better understanding of farmers’ fungicide spraying behavior in the context of varying wheat rust pressure levels. This is crucial to enhance the effectiveness and efficiency of wheat rust management in Ethiopia and other wheat producing countries. The paper concludes by discussing potential entry points for enhancing site-specificity of information on wheat rust predictions to farmers.

## Results

### Moderate wheat yellow and stem rust pressure levels during 2022/23 growing season

Altitude, temperature, and moisture strongly influence infection prevalence and severity of wheat stem and yellow rust. However, the expansion of irrigated wheat can facilitate the movement of stem rust races within the country and across borders^23^. We assessed predicted wheat yellow and stem rust pressures for ten districts in the Amhara, and eight districts in the Oromia regions (Figure S1).

**Fig. 1:**
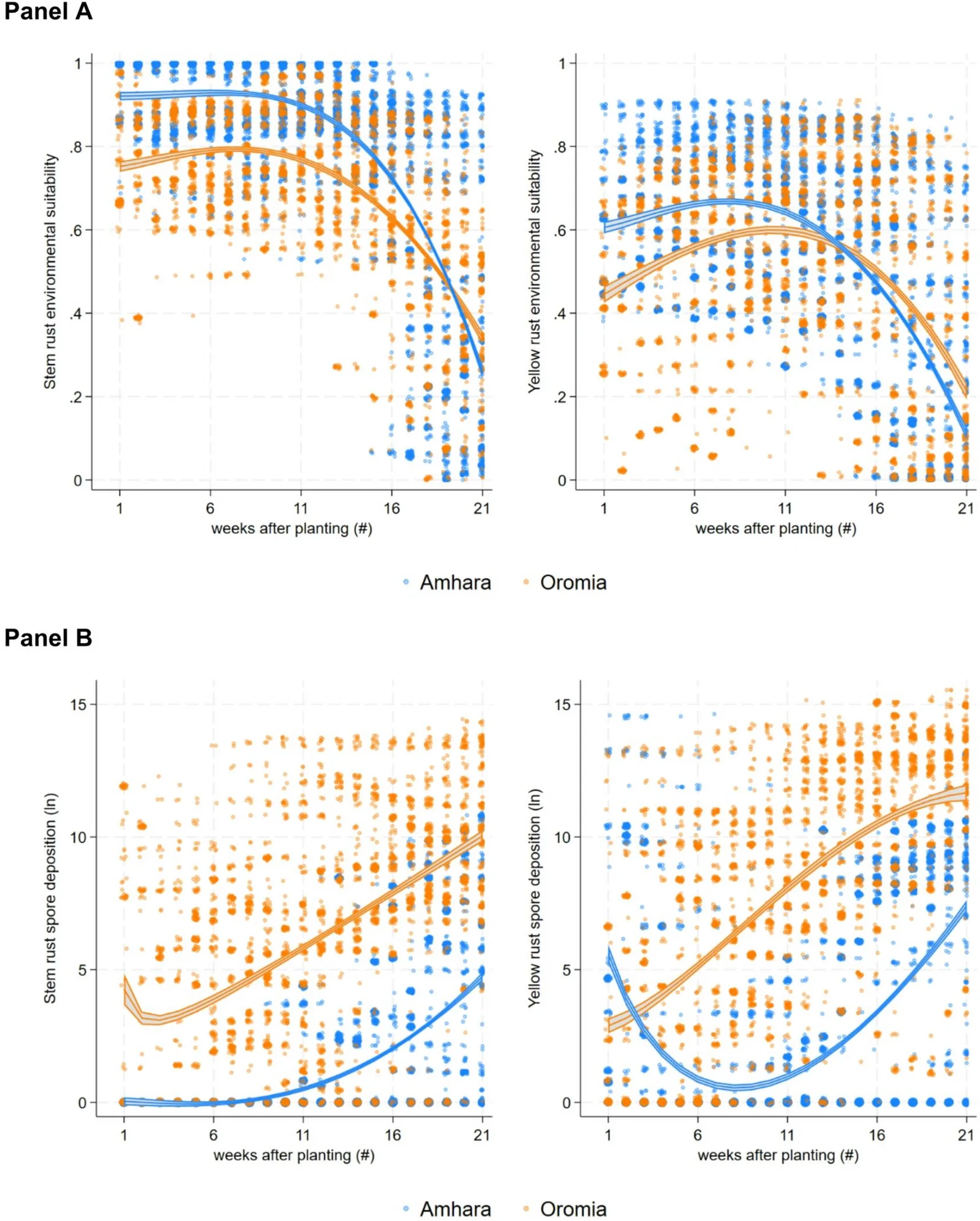
Predicted environmental suitability (Panel A) and predicted spore deposition (Panel B) of stem and yellow rust by week after planting for the 2022/23 growing season. Notes: Panels show scatterplots and fractional polynomial fitted curves. One data point presents one wheat plot at the respective week after planting. The total number of data points is *N* = 10,179. Predicted environmental suitability is expressed as an index from 0–1 with 1 resembling optimal environmental condition for spore development. Predicted spore deposition is the natural log transformed number of rust spores per square meter per three hours. Data points with values below 1, which indicate a very low predicted spore deposition level, were set to 0 prior to the natural log transformation. Predictions for environmental suitability and spore deposition were made at the Woreda level. Source: Survey data. Figure created with STATA software (version 18).

For the 2022/23 main cropping season, the EWAS forecasts indicated moderate yellow and stem rust pressure levels. Predicted environmental suitability for both stem and yellow rust followed similar temporal patterns over the growing season across sampled districts in the Amhara and Oromia regions, with higher suitability for stem rust than for yellow rust in both regions. Within the growing season, predicted environmental conditions for stem and yellow rust development were most favorable between six to eleven weeks after planting and declined with the progressing growing season (Fig. 1, Panel A). Predicted spore deposition levels for stem and yellow rust in sampled districts in the Oromia region were generally higher compared with sampled districts in the Amhara region throughout the growing season (Fig. 1, Panel B). Sampled farms from the Oromia region tended to have wheat plots at lower altitudes compared with sampled farms in the Amhara region.

### Farmers’ wheat rust management through chemical control measures

The use of chemical control measures can be an effective within-season strategy to mitigate wheat rust infections and related production losses^12^. Our data indicate that most farmers have used fungicides in wheat production in past seasons (Table 1). The application of fungicides was much more prevalent among interviewed farmers in Oromia compared with interviewed farmers in the Amhara region. Sampled wheat producers in Oromia also applied significantly more fungicides per wheat area during the 2015-20 seasons than sampled farmers in Amhara. This disparity was also observed for the 2022/23 growing season, during which the share of fungicide users, the applied quantities, and number of fungicide applications were significantly larger for sampled wheat farmers in Oromia.

**Table 1:** Farmers’ fungicide use and predicted wheat stem and yellow rust pressure levels by region.

	Total (*n* = 520)		Amhara (*n* = 312)		Oromia (*n* = 208)	
	Mean	SD	Mean	SD	Mean	SD
Fungicide use in past seasons						
Proportion of farmers who have previously used fungicides	0.58	0.49	0.30^***^	0.46	1	0
Year of first fungicide use^#^	2007	5.15	2010^***^	4.28	2006	5.09
Average fungicide use between 2015-20 (liter/ha)	0.56	0.53	0.19^***^	0.33	1.11	0.15
Proportion of farmers who have used fungicides in 2021/22 season	0.50	0.50	0.19^***^	0.39	0.97	0.17
Fungicide use in 2022/23 season						
Proportion of farmers who have used fungicides in 2022/23 season	0.61	0.49	0.36^***^	0.48	0.99	0.07
Quantity of fungicides used in 2022/23 season (liter/ha)^##^	1.27	1.80	0.49^***^	1.00	2.42	2.08
Number of fungicide applications in 2022/23 season	1.73	1.64	0.61^***^	0.92	3.43	0.81
Stem rust in 2022/23 season						
Mean predicted spore deposition until 121 days after planting (ln)	4.67	4.02	2.00^***^	2.64	8.67	1.81
Mean predicted environmental suitability until 121 days after planting (0–1)	0.82	0.09	0.87^***^	0.06	0.74	0.08
Yellow rust in 2022/23 season						
Mean predicted spore deposition until 121 days after planting (ln)	7.39	4.16	5.40^***^	3.82	10.38	2.56
Mean predicted environmental suitability until 121 days after planting (0–1)	0.60	0.17	0.63^***^	0.16	0.56	0.17

Notes: ^***^ indicates mean values of Amhara and Oromia region are significantly different from zero at the 1% significance level. # for users only. ## outliers (> 99%tile) removed.

For this study, we specified the beginning and end of the critical growth stage for wheat based on plot specific altitude levels which we combined with the wheat growth and phenology model developed by Acevedo et al.^24^ for growing environments in Ethiopia^25^.

**Fig. 2:**
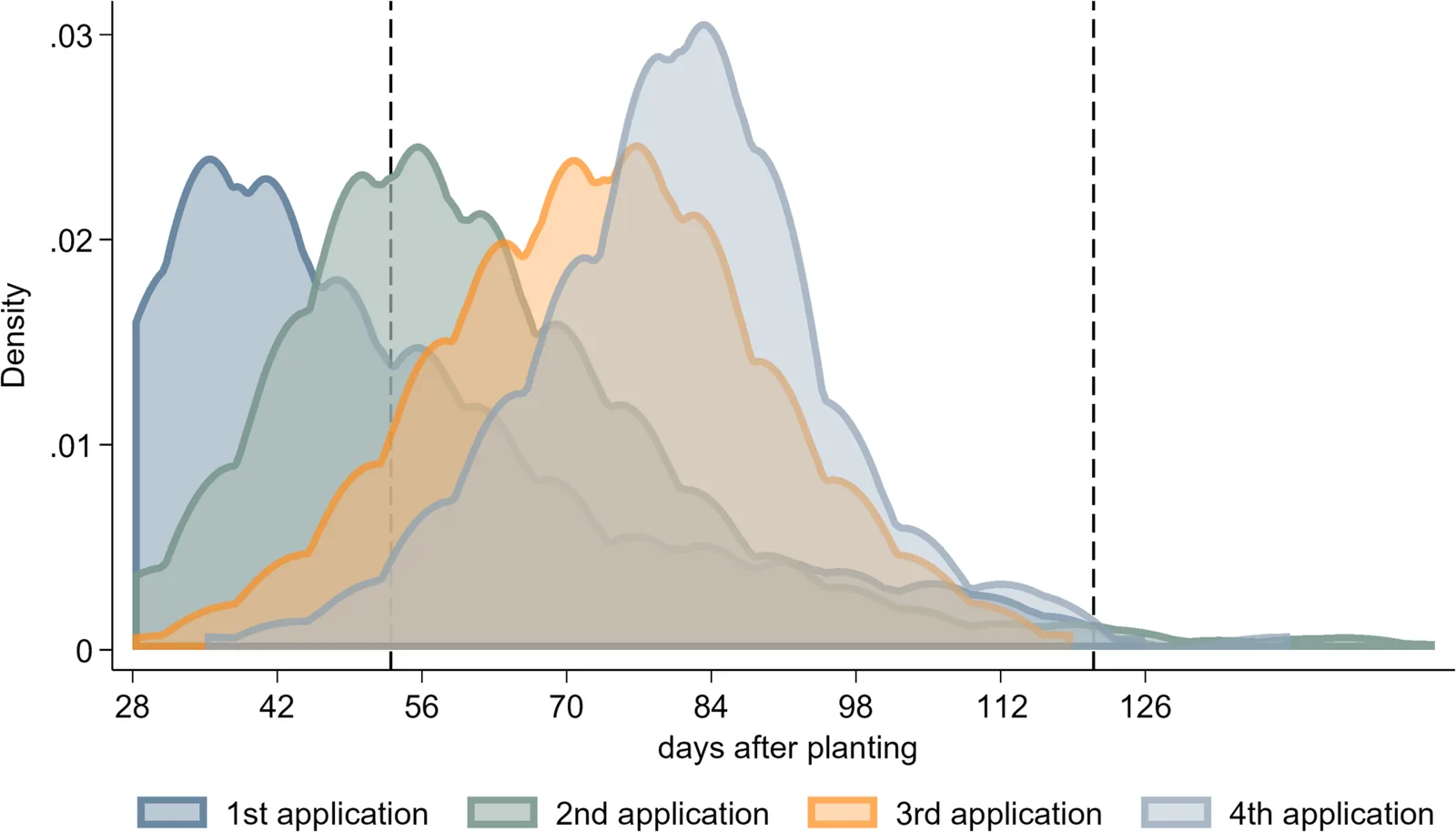
Kernel density estimates of the timing of fungicide applications expressed in days after planting in the 2022/23 wheat-growing season by sample farmers. Notes: Dashed lines indicate the start and end of susceptible growth stages of wheat (53–121 days after planting) during which wheat is most vulnerable to stem and yellow rust. The start corresponds to the lowest altitude (< 2000 meter above sea level, m abs.), the end to the highest altitude (> 2600 m abs). Number of observations: *n* = 313 (1st application); *n* = 265 (2nd application); *n* = 203 (3rd application); *n* = 84 (4th application). Applications within the first 28 days after planting were excluded. Source: Household survey data. Figure created with STATA software (version 18).

Our data shows that there was a large variation in the timing of fungicide applications. Around 23% of applications occurred before the start of critical growth stages of wheat (Fig. 2).

### Wheat rust information reached most farmers

Forecasts of the EWAS are shared with national partners and transmitted to farmers via the national agricultural extension system. Extension agents provide farmers with time and location-specific information on predicted wheat rust pressure levels to support their rust management decisions. Our results show that wheat rust information reached 73% of sampled wheat producers during the 2022/23 growing season (Table 2). EWAS messages are mainly disseminated through agricultural extension systems and are not targeted to specific farmers. Full coverage is therefore not expected. Information diffusion was relatively wider among sampled farmers in the Amhara region compared with sampled farmers in the Oromia region. In both regions, the most important transmission channels were agricultural extension officers who operate at the *Woreda* (District) level under the supervision of the Regional Bureaus of Agriculture. Dissemination of information on wheat rust development through extension agents can largely be attributed to the EWAS. Beyond official channels, farmers also relied on neighboring farmers and radio programs for wheat rust information. Both channels were relatively more important among interviewed farmers in the Oromia region. Due to modest rust pressure levels in the 2022/23 season, no phone-based warnings were emitted, which are only applied in high-risk scenarios. About 90% of farmers rated the information to have been useful when determining the timing and intensity of fungicide applications which underlines the degree of appreciation farmers had for wheat rust related advisories.

**Table 2:** Diffusion of wheat rust information to sample farmers in 2022/23 season.

	Total (*n* = 520)	Amhara (*n* = 312)	Oromia (*n* = 208)
	Mean	Mean	Mean
Proportion of farmers who received wheat rust information	0.73	0.78^***^	0.67
Frequency of information exposure *(no.)* (sd.)	2.02 (1.55)	1.98 (1.42)	2.06 (1.72)
Proportion of farmers who received information through^#^…			
Extension officer	0.94	0.93	0.95
Neighbor	0.32	0.28^**^	0.40
Radio	0.21	0.17^***^	0.28
Phone	0.02	0.02	0.02
Proportion of fungicide users who used information to guide…			
Timing of fungicide application	0.89	0.84^***^	0.94
Quantity of applied fungicides	0.90	0.84^**^	0.94

Notes: ^#^ for information recipients only. ^***^, ^**^ indicate that differences in proportions between the Amhara and Oromia regions are significantly different from zero at the 1 and 5% significance level, respectively.

Farmers’ information exposure was not assigned randomly, but was likely determined by farm and household characteristics, such as farming experience, level of education, participation in village-level institutions, and farm size^26,27^. We modeled the influence of predicted stem and yellow rust environmental suitability and spore deposition levels on the probability of information reception at the farm level (Table 3). Since we did not have information on the time of farmer information exposure in the growing season, we focused on yellow and stem rust pressure predictions during the first 53 days after planting. We do so as we expect information to be diffused rather early in the season, i.e., before wheat enters yellow and stem rust susceptible growth stages.

Estimation results indicate that information diffusion to farmers was associated with predicted favorable environmental conditions for stem and yellow rust development. More suitable predicted environmental conditions for stem and yellow rust during the first 53 days of the growing season increased the probability that a household received wheat rust related information (Model 1), and that it received information multiple times during the season (Model 2). Results remain robust when we controlled for additional farm and household level characteristics such as respondent age and education, household size, farmer organization membership, wheat cultivation area, and past exposure to rust information (Table S1).

**Table 3:** Correlates of farmers’ exposure to wheat rust information.

	Model (1)	Model (2)
	Farmer received wheat rust information (yes/no)	Frequency of wheat rust information exposure (no. of times)
Stem rust		
Spore deposition 1–53 days after planting *(natural log)*	0.01 (0.01)	0.14*** (0.04)
Environmental suitability 1–53 days after planting (0–1)	0.81** (0.35)	4.06** (1.76)
Yellow rust		
Spore deposition 1–53 days after planting *(natural log)*	0.01 (0.01)	0.05 (0.04)
Environmental suitability 1–53 days after planting (0–1)	0.58*** (0.19)	3.05*** (0.85)
No. observations	462	462
Pseudo *R*^2^	0.09	0.03
Log pseudo-likelihood	−239.0	−825.04
Wald χ^2^ (4)	32.82	
F statistic		5.96

Notes: Estimates are marginal effects, showing how the probability of the outcome changes with a one-unit change in the independent variable. Model 1 is a Probit model, with the dependent variable indicating whether the farmer received wheat rust information in the 2022/23 season (1 = yes, 0 = otherwise). Model 2 is a Tobit model, with the dependent variable representing the frequency of exposure to wheat rust information (right-censored: 0, 1, …, n). Predicted rust spore deposition and environmental suitability are averaged over the first 53 days after planting. Standard errors (in parentheses) are robust and clustered at the district level. **, *** denote significance at the 5%, and 1% levels, respectively. The number of observations is less than 520 due to missing data for 58 observations, i.e., for observations that have planted wheat before the start of weekly rust pressure predictions.

### Regional and altitude-based differences in farmer fungicide use

**Fig. 3:**
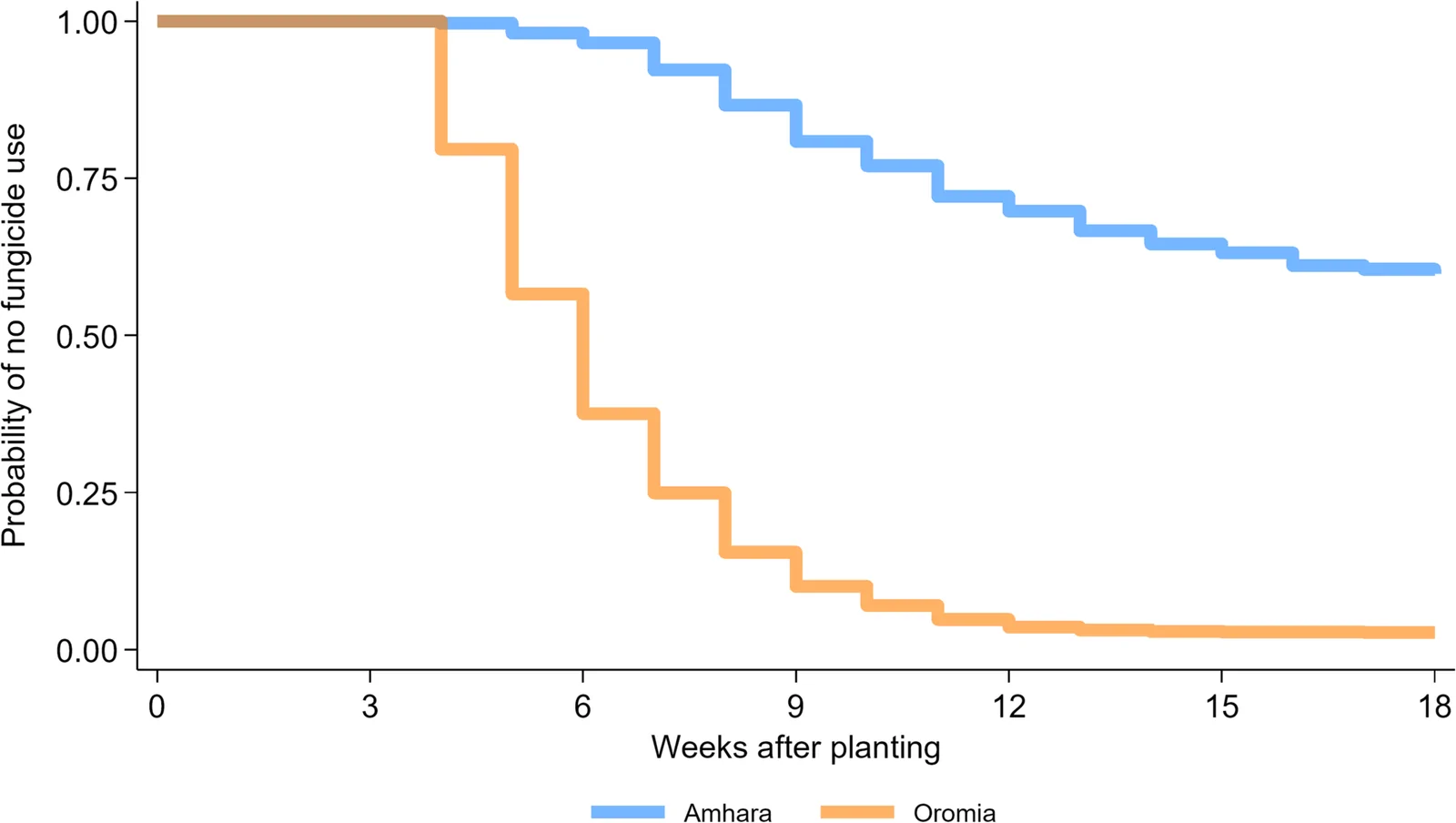
Kaplan-Meier survival estimates by region for time to initial fungicide use. Notes: The Kaplan-Meier estimates are based on duration analysis. They make no assumption about the underlying distribution of times to fungicide use. The survival estimate for a given period is derived by dividing the number of households that have not used fungicides in the respective week after planting by the total number of households during the same period^28^. Number of observations: 520. Source: Household survey data. Figure created with STATA software (version 18).

When assessing fungicide use during the growing season, we found a significant regional difference in time to first spray after planting (Fig. 3). More than 50% of sampled farms from the Oromia region had applied fungicides at least once during four to five weeks after planting, while there were practically no applications among sampled farmers in the Amhara region.

**Fig. 4:**
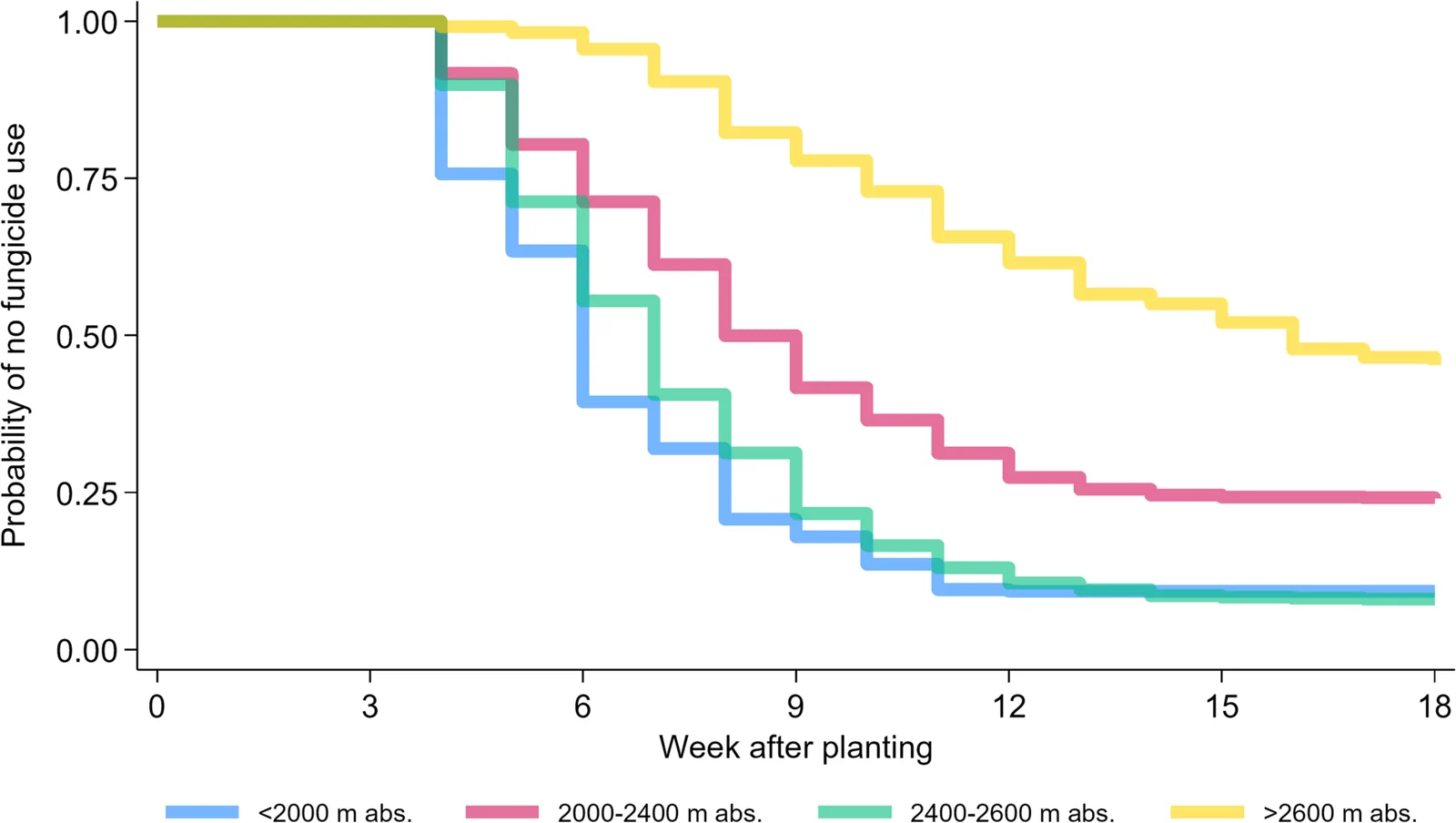
Kaplan-Meier survival estimates for time to initial fungicide use at different farm altitudes. Notes: See notes for Fig. 3. Observation specific farm level altitudes were used to assign observations to altitude categories. Number of observations: 520. Source: Household survey data. Figure created with STATA software (version 18).

When time spells from planting until fungicide use were disaggregated by farm-specific elevation, we found that farmers tended to spray sooner in wheat plots at lower altitudes (Fig. 4).

### Wheat rust pressure levels and farmers’ exposure to advisory services are associated with fungicide use, and timely fungicide use

We next examine fungicide use over the 2022/23 growing season (Table 4, Model 1). Predicted yellow rust spore deposition was significantly associated with the timing of fungicide applications. Jointly interpreting the main effect of predicted yellow rust spore deposition and its interaction with growing week indicates that hazard ratios for fungicide application remained positive until week 10 after planting as predicted levels of yellow rust deposition increase (see table notes for interpretation of hazard ratios). In other words, higher predicted yellow rust pressure levels were associated with earlier fungicide use between weeks four to week ten after planting. Predicted environmental suitability for yellow rust had a positive effect on the use of fungicide applications in the later stages of the growing season, i.e., from week 10 after planting. In contrast, we did not find significant associations between predicted stem rust deposition or environmental suitability and farmer fungicide application timing in this model specification. These results hold when controlling for farm size, past fungicide use, and agro-ecological variation through altitude categories and their interaction with time after planting.

Farmers’ exposure to wheat rust information was positively associated with earlier fungicide application. According to estimated hazard ratios, each additional exposure to wheat rust information increased the rate of fungicide use by 9–11% in any week after planting. This association remained statistically significant when controlling for predicted rust pressure, environmental suitability, and other covariates included in the specification.

The timing of fungicide applications during the season was also positively associated with farm and household characteristics. Farmers with prior experience of fungicide use exhibited a 21–22% higher hazard of application at any given week after planting. Larger wheat area cultivated was associated with a higher hazard of fungicide use earlier in the season. In addition, altitude was associated with application timing, with farms at lower altitudes exhibiting higher hazards of earlier fungicides applications, and farms at higher altitudes exhibiting lower hazards over the same period. These associations were estimated conditional on the full set of covariates included in the model.

We also assessed timing of fungicide application during critical growth stages of wheat (Table 4, Model 2). We found that an increase in predicted yellow rust pressure was associated with delayed fungicide use, while more favorable predictions for environmental suitability for yellow rust led to significant earlier application during the critical growth stages. In contrast to the results for yellow rust, predicted stem rust spore deposition and environmental suitability were not significantly associated with fungicide application timing in this specification. Farmer exposure to wheat rust information remained positively associated with fungicide use, with each additional exposure increasing the hazard of application by about 11%. These estimates were derived from specifications that controlled for farm size, past fungicide use, and agro-ecological conditions across both study regions.

**Table 4:** Duration analysis of associations between rust pressure levels, information exposure and fungicides use.

	Model (1)		Model (2)	
	Use of fungicides in growing season (weeks 4–18 after planting)		Use of fungicides in critical growth stages of wheat (plot altitude specific time window)	
	Coefficients	Hazard ratios	Coefficients	Hazard ratios
Stem rust spore deposition *(ln)*	0.01 (0.01)	1.01	0.01 (0.02)	1.01
Stem rust spore deposition (ln) * week after planting (wap) (interaction)	−0.01 (0.01)	0.99		
Yellow rust spore deposition (ln)	0.06^***^ (0.04)	1.07^***^	−0.08^***^ (0.02)	0.92^***^
Yellow rust spore deposition (ln) * week after planting (wap) (interaction)	−0.01^***^ (0.01)	0.99^***^	-	-
Stem rust environmental suitability (0–1)	0.18 (0.90)	1.20	0.30 (0.46)	1.34
Stem rust environmental suitability * wap (interaction)	0.01 (0.10)	1.01	-	-
Yellow rust environmental suitability (0–1)	−1.22^***^ (0.66)	0.29^***^	0.86^**^ (0.35)	2.36^**^
Yellow rust environmental suitability * wap (interaction)	0.21^***^ (0.08)	1.23^***^	-	-
Farmer received wheat rust information (no. information exposures)	0.09^***^ (0.01)	1.09^***^	0.11^***^ (0.03)	1.11^***^
Cultivated wheat area in 2022/23 season (ha)	0.02^**^ (0.01)	1.02^**^	−0.03 (0.03)	0.97
Farmer is a regular user of fungicides (dummy)	0.19^***^ (0.06)	1.21^***^	0.20^*^ (0.12)	1.22^*^
Farm altitude category (< 2000; 2000–2400; 2400–2600; >2600)	−0.67^***^ (0.19)	0.51^***^	0.01 (0.09)	1.00
Farm altitude category * wap (interaction)	0.08^***^ (0.02)	1.08^***^	-	-
Number of subjects (farmers)	452		452	
Number of failures (fungicide applications)	726		313	
Observations	6,462		2,325	
Log-pseudolikelihood	−3847.57		−1567.86	
Wald chi^2^	124.88		41.10	

Notes: Robust standard errors in parentheses. Hazard ratios are exponentiated coefficients; values larger (smaller) than 1 indicate faster (slower) time to fungicide use. *, **, *** indicate significance are the10%, 5% and 1% levels, testing if coefficients are equal to zero and hazard ratios equal to unity. In model 1, the outcome is whether a farmer applied fungicides during weeks 4 to 18 after planting. In model 2, the outcome is whether a farmer applied fungicides during the critical growth stages of wheat rust infestation, which are altitude specific (see methods). Time-variant variables are predicted yellow and stem rust spore deposition and environmental suitability. Time-invariant covariates are farmer exposure to wheat rust information in the 2021/22 season, farmer regular use of fungicides, wheat cultivation area in the 2022/23 season, farm altitude, and number of times the farmer was exposed to wheat rust information in the 2022/23 season. Additional covariates (not shown) are farmer age and education level, household size, and membership in village-level agricultural institutions. Due to hazard non-proportionality, predicted stem and yellow rust spore deposition and environmental suitability, as well as farm altitude category were interacted with the duration of the time spell in Model 1. Aggregated covariate effects are computed as: main effect*interaction effect^time spell^.

## Discussion

Wheat rusts continue to be severe threats to wheat production in Ethiopia. In response to past epidemics, breeding targets have shifted towards incorporating rust resistance traits into newly released varieties. While varieties with wheat rust resistance traits are increasingly adopted by smallholder farmers^13^, the incursion of new rust races exerts continuous pressure on genetic resistance of wheat cultivars^7^. In this context, farmers need to rely on the use of fungicides within the growing season to combat wheat rust infection^12^. The wheat rust Early Warning and Advisory System (EWAS), which was piloted from 2015 and deployed in Ethiopia since 2017, provides summaries of disease status from field surveys and forecasts of the risk of infection. The EWAS was designed to enhance farmers’ wheat rust management. It also has an added benefit of informing national planning of fungicide imports and distribution. Enhancing and optimizing the effectiveness of fungicide applications in crop production can safeguard economic resources, both at the national and at the farm level, increase farmers’ crop incomes, and reduce negative impacts on human and ecosystem health associated with the application of chemical agents for disease control.

Across the sample, a substantial share of farmers reported using fungicides during the growing season, with many applying them more than once. Notably, approximately one fourth of fungicide applications occurred early in the season, prior to the growth stages at which chemical control is typically considered most effective for protecting yield-forming plant tissues^29^. This pattern suggests that fungicide use is not only guided by crop development stages, but also reflects precautionary strategies shaped by past experiences with rust outbreaks. Similar preventive approaches have been documented in other wheat-growing regions, where early applications are used to mitigate perceived risk of disease build-up^30^ and spread^31^. More broadly, such behavior may reflect farmers’ expectations regarding the rust pressure development based on experience, which have been shown to influence agricultural decision-making in other contexts^32^^33^.

Beyond these general spraying behaviors, fungicide use among sample farmers in Oromia was nearly universal. This descriptive difference between the sampled locations should not be interpreted as an effect of regional administrative classification. Rather, it may reflect differences among the sampled districts in fungicide access, historical exposure to rust outbreaks, and agroecological conditions affecting disease pressure. From 2010 to 2019, the sampled districts in Oromia experienced higher stem rust prevalence compared with sampled districts in Amhara^7^, which may have contributed to the adoption of more preventive spraying strategies earlier in the growing season. These differences are likely driven by local epidemiological and agroecological conditions and do not imply uniformly higher rust pressure or fungicide use across Oromia. More frequent and intensive applications in the sampled locations may therefore reflect a response that has become embedded in local management practices. Differences in fungicide timing across locations may also reflect agro-ecological conditions: since wheat development slows with increasing altitude and lower mean temperatures^24^, later applications would be expected in higher-elevation areas, reflecting shifts in the timing of critical growth stages.

While these descriptive patterns highlight variation in farmer spraying behavior across regions and agro-ecological conditions, the empirical analysis enables a more systematic assessment of how predicted rust pressure, environmental suitability, and information exposure shaped fungicide use and its timing. Our results indicate that these factors were associated with the timing of farmer fungicide applications during the season, although the strength and direction of these associations varied across factors and rust types.

Predicted yellow rust spore deposition was associated with earlier fungicide applications, whereas predicted environmental suitability for yellow rust was associated with accelerated fungicide use during later stages of the growing season. This later period corresponds to wheat development stages between jointing and flowering, during which fungicide applications are generally most effective in protecting yield-forming plant tissue^34^.

In contrast, we did not find significant associations between predicted stem rust pressure or environmental suitability and timing of fungicide applications. This suggests that stem rust risk may have been less consistently translated into fungicide use decisions across locations, or that responses varied in ways that attenuated average effects in the pooled sample.

Farmers’ exposure to wheat rust information was consistently associated with earlier fungicide application, including during critical growth stages. However, the persistence of precautionary spraying behavior suggests that farmer decision-making was not solely driven by advisory information. While such behavior may reduce the likelihood of severe yield losses, it may also limit the efficiency of fungicide use when applications are not optimally timed.

Overall, these findings highlight that the timing of fungicide use was associated with biophysical signals of disease risk and farmers’ access to and interpretation of advisory information. While the EWAS appeared to support earlier and more responsive fungicide use, the persistence of early and potentially inefficient applications indicates scope to further improve the targeting and timing of advisory messages, particularly in relation to crop growth stages, local agro-ecological conditions, and rust-specific dynamics.

Our findings suggest that location- and time-specific messaging on disease risk may contribute to improved wheat rust management. However, several limitations should be considered when interpreting these results. First, information exposure is measured as a static variable and does not capture the timing of advisory receipt within the season, which may influence its association with fungicide use. Second, we do not observe the specific content or mode of information transmission between extension agents and farmers, which may affect how advisory messages are interpreted and acted upon. Third, disruptions to field surveillance in parts of the study area may have affected the completeness of EWAS-based predictions of rust pressure. While these limitations constrain causal interpretation, the data cover key aspects of interest and allow for the modeling of associations between predicted rust pressure, information exposure, and fungicide use.

In this context, our findings indicate that most farmers made use of wheat rust information to guide the timing and intensity of fungicide applications. At the same time, the persistence of early and potentially sub-optimal applications suggests scope to further improve the effectiveness of advisory systems. First, the EWAS could enhance the targeting and customization of advisory messages to better reflect local conditions. Incorporating farm-level characteristics, such as planting dates, and varietal susceptibility could support more tailored and timely recommendations. Such an approach may improve alignment between advisory messages and crop development stages and help reduce unnecessary fungicide applications in low-risk environments. The rapid expansion of mobile phone connectivity in Ethiopia - reaching roughly 64% of the population in 2025 - provides an important foundation for mobile-based EWAS advisory delivery, although disparities in access may still limit coverage in some regions^35^.

Second, the EWAS could be further developed to address a broader range of wheat diseases and management challenges. Expanding the system to include emerging threats, such as Fusarium head blight, would require more precise and time-sensitive advisory capabilities, but could enhance the overall responsiveness and relevance of the system for farmers and policymakers.

Overall, strengthening the integration of localized data and real-time advisory messaging has the potential to improve the efficiency and effectiveness of disease management in wheat systems. By supporting more targeted and timely fungicide use, such developments could contribute to more resilient production systems, improved farmer livelihoods, and reduced environmental impacts.

## Methods

### Database and data collection

The study relied on a sample of wheat farmers surveyed by Mottaleb et al.^11^. during the 2020/21 season. The original survey was conducted in November and December 2020 in 17 purposively selected wheat-producing districts: Basoliben, Motta, Wonberima, Burie, Lay Gaint, Misrak Estie, Mekiet, Wadila, and Debay Telatgin in Amhara; and Arsi Robe, Hitosa, Gedeb Assosa, Dodola, Sinana, Agarfa, Ginir, and Golocha in Oromia. The districts were selected to cover important and contrasting wheat-production systems, including differences in historical exposure to yellow and stem rust, production potential, and access to extension and agricultural inputs. Within each district, farmers were selected from a district-level sampling frame, with an initial target of 76 wheat farmers per district. The original survey included 1,067 farmers, of whom 724 had active telephone numbers and constituted the sampling frame for the present study. Both regions account for over 80% of the total national wheat growing area and production^6^. Ethical approval for this study was granted by the CIMMYT Internal Research Ethics Committee (IREC; application number IREC.2022.040), after which 526 farmers were successfully interviewed during the 2022/23 wheat-growing season (Table S2 and Figure S1). The main wheat growing season in Ethiopia (Meher season) typically runs from June/July to November/December, with some variation across regions and elevations.

Each farmer was interviewed three times during the season using phone surveys. A baseline interview was carried out shortly after wheat planting, a midterm interview was conducted during the second half of the growing season, and an endline interview was implemented after harvest. The surveys collected data on the main farm characteristics and wheat production. Farmers were asked to identify their most important wheat plot, for which sowing dates and the timing of fungicide applications were recorded. The survey also included sections on farmer exposure to wheat rust information, past use of fungicide, and access to fungicides in the current season.

Several steps were taken to ensure data quality and completeness. The questionnaire was pre-tested and administered by trained enumerators using the dominant local languages (Amharic or Afan Oromo). Repeated interviews within the same season enabled cross-checking of key responses; later rounds incorporated information from earlier interviews to ensure consistency in plot identification and the timing of fungicide applications, thereby improving data reliability and reducing recall bias. No additional data from key informants were used, as the analysis focused on farmer-level decision-making.

While phone surveys offer logistical advantages, they may introduce potential sources of bias. In particular, coverage bias may arise if mobile phone access is correlated with key variables of interest, and non-response may affect sample representativeness if respondents differ systematically from non-respondents^36^^37^. To mitigate these risks, multiple call attempts were made at different times of the day, and interviews were kept short to reduce respondent burden. The overall response rate was 73%, which is comparable to phone surveys conducted in similar contexts^37^. Nonetheless, some degree of non-coverage and non-response bias may remain, potentially leading to underrepresentation of poorer and less connected households^36^.

### The Early Warning and Advisory System

The EWAS is designed and operated by a group of national and international partners, including the University of Cambridge, the U.K. Met Office, the Ethiopian Institute of Agriculture Research (EIAR), the Ethiopian Agricultural Transformation Institute (ATI), and the International Maize and Wheat Improvement Center (CIMMYT)^10^. The system underwent a pilot study in the 2015–2016 seasons and was deployed in 2017. The EWAS is supported by a numerical dynamical modelling system that forecasts the risks of infection for the upcoming week^10^^38^. The EWAS receives input data on the current status of stem and yellow rust in wheat-growing areas of Ethiopia. These data are obtained from field surveys carried out by trained plant pathologists, with data automatically uploaded using Open Data Kit software (https://getodk.org/). Meteorological forecast data for seven days beyond the date of predictions are obtained from the UK Met Office Unified Model^39^. The EWAS forecasts consist of three model components comprising a model for source calculation, a Lagrangian model for spore dispersion and deposition (an adapted version of the UK Met Office’s particle dispersion model NAME^40^), and an environmental suitability model for infection. The source calculation model processes the disease surveys, generates representative source locations, and calculates source strength in terms of spores available for release at each representative location. The adapted NAME model simulates the weather-dependent spore release, Lagrangian aerial transport, UV decay of the spores, and the deposition (wet and dry) of the viable spores^41^. The environmental suitability model uses a set of criteria to predict the probability of successful infection at spore landing sites. The combined EWAS models output risk maps for spore deposition and environmental suitability for infection, across all wheat-growing areas in Ethiopia. The maps are resolved to 0.09375 degrees N-S by 0.140625 degrees E-W (approximately 11 km x 16 km at the equator), determined by the resolution of the meteorological forecast data.

The forecast outputs together with field survey statistics are reviewed by trained experts who produce weekly wheat rust advisories. The advisories provide a summary and recommendations along with risk maps that are cascaded to extension agents and smallholder farmers through government agencies. For the purposes of this study, the 1-day forecasts for the 2022-23 rain-fed seasons were averaged weekly and by *Woreda*.

### Defining critical growth stages of wheat

The aim of chemical control measures is protecting critical plant tissues from infection, i.e., the flag leaf and the second leaf which are most important for producing sugars for grain development^31^. Achieving this protection depends on optimizing the timing of fungicide applications. Fungicide timing can be guided by infection threshold on plant tissues or individual plants, plant growth stage, or preventive application strategies^29^. While the most effective time to apply fungicides is considered to be between last leaf emergence (GS 39) and complete head emergence (GS 59)^34^, preventive sprays can be recommended as early as the beginning of stem elongation (GS 30). The time to reach these growth stages depends on the climatic conditions as well as on the type of variety (early, medium and late maturing).

In Ethiopia, wheat-growing environments were categorized into four altitude ranges (meter above sea level, m abs) based on the incidence and distribution of wheat yellow and stem rust^42^: low (< 2000 m abs), medium [2000–2400 m abs), mid-high [2400–2600 m abs) and high (> 2600 m abs). Using the wheat growth and phenology model by Acevedo et al.^24^, the duration of wheat development stages from germination to maturity was estimated for these altitudes based on data generated for the work of Badebo^25^, although not all are reported in the written thesis. We defined critical wheat growth stages as the period from jointing to heading and derived altitude-specific time windows covering the various maturing rates of wheat varieties: 53–75 days for altitudes < 2000 m asl; 62–87 days for 2000–2400 m asl; 69–97 days for 2400–2500 m asl; and 86–121 days for > 2600 m asl.

### Duration analysis

Duration models have their origin in biomedical sciences and industrial engineering^43^. Their focus is on explaining the length of a spell of non-occurrence of an event of interest. In the setting of this study, the length of the spell refers to non-use of fungicides. This probability is reflected by the hazard rate (λ), which is the core function in duration analysis^44^:

$$\:\lambda\:\left(t\right)=\underset{\varDelta\:\to\:0}{\mathrm{lim}}\frac{\mathrm{Pr}\left(t+\varDelta\:t>T\right|T>t)}{\varDelta\:t}$$

With *T* denoting a non-negative random variable, and *t*, a realization of *T*, representing the duration in a specific state (e.g., non-use of fungicides). The hazard rate is also known as the instantaneous rate at which a spell ends at duration *T* = *t*, given that it has lasted until *t*. The hazard rate can be interpreted as the limiting probability that the event of interest occurs at a given point, conditional upon non-occurrence of the event until that point.

We used this framework to explain the duration until usage of fungicides within the growing season 2022/23. In this context, we set our data in a time-discrete manner. For each farmer, one time spell corresponds to one week of the growing season, starting with the individual week of planting and ending in the week of the first fungicide application. If a farmer used fungicides multiple times in the season, the spell restarts in the week after the application and ends in the week of the subsequent application, or at 22 weeks after planting, after which stem and yellow rust management become practically irrelevant. If a farmer did not use fungicides, the spell ends at week 22 after planting.

As explanatory variables, we included predicted yellow and stem rust spore deposition, predicted yellow and stem rust environmental suitability, the number of farmer exposures to wheat rust information in the 2022/23 season, whether the farmer was a regular user of fungicides, the total farm area under wheat cultivation in the 2022/23 season, farm altitude, farmer age and education level, household size, and whether the farmer was a member in village-level agricultural institutions. In duration analysis, covariates are either time-variant, i.e., changing their values over time spells, or time-invariant. Predicted rust deposition and environmental suitability data were time-variant, while all other variables were time-invariant in our models.

We expected that location-specific factors may affect the time to fungicide use within a season. While we assumed that predictions of rust spore deposition and environmental suitability were good predictors of fungicide demand from producers, we do not have data on fungicide supply and availability, which may lead to omitted variable bias^46^. The use of static location dummies could not mediate this shortcoming in a dynamic modeling framework. One approach is the estimation of duration models with shared frailties, in which observations from the same group share the same frailty. In this sense, frailties are analogous to random effects in linear regression models^45^. We set shared frailties at the region level. When estimating the time to fungicide use, we used the extended Cox-model^44^, which leaves the underlying baseline hazard unspecified and enables the testing of the proportional hazards assumption. Proportional hazard models assume that covariate effects are time independent, i.e., the effect of covariates on the hazard ratio remains unchanged over the time spell under analysis^45^. We found evidence for non-proportionality, which we corrected by including time spell interactions^47^. Accordingly, the hazard rate in the fungicide use models is formally estimated as:

$$\:{\lambda\:}_{ij}\left(t|{x}_{i},{\gamma\:}_{j}\right)=\:{\lambda\:}_{0\:}*\mathrm{e}\mathrm{x}\mathrm{p}({x}_{i}{\beta\:}_{m}+\left({x}_{i}*{t}_{i}\right){\beta\:}_{t}+log{\gamma\:}_{j})$$

With $\:{\lambda\:}_{ij}\:$indicating the hazard ratio for farmer *i* in region *j*,* t* is the duration expressed in growth weeks, $\:{x}_{i}$ is the vector of the introduced explanatory variables, $\:{\gamma\:}_{j}$ are shared frailties in region *j*, and $\:{\beta\:}_{m}$ and $\:{\beta\:}_{t}$ are the covariates main and time spell interaction effects (if applicable) which are to be estimated from the data.

One limitation of our data is that we do not have information on the exact time of information reception during the season. However, around 90% of farmers rated the wheat rust information to be useful when determining the timing and intensity of fungicide applications. We therefore expect farmers’ first information exposure to have occurred mostly before their first fungicide application. Additionally, approximately 40% of farmers reported receiving information only once during the season, and around 50% applied fungicides only once. In such cases, the timing of information appears to have limited relevance for the duration analysis. Nonetheless, our models did not assess time-specific effects of information exposure on fungicide use during different growing weeks.

## Supplementary Information

Below is the link to the electronic supplementary material.

Supplementary Material 1

## Data Availability

Household survey data and data from the EWAS model are available at a reasonable request. The household survey data will be shared in accordance with CIMMYT’s data sharing policies and subject to any applicable ethical or confidentiality constraints.
