# Supplementary material for: Early warning and advisory can contribute to enhanced wheat stem and yellow rust management in Ethiopia
Source: Sci Rep. 2026 Aug 14;16:37911. doi: 10.1038/s41598-026-67016-9 (PMC13759324; doi:10.1038/s41598-026-67016-9)
Supplement: Supplementary file 1 [file 41598_2026_67016_MOESM1_ESM.docx]

**Supplementary Materials**

**Table S1**. Correlates of farmers’ exposure to wheat rust information with additional farm and household control variables.

|  | Model (1) | Model (2) |
| --- | --- | --- |
|  | Farmer received wheat rust information  *(yes/no)* | Frequency of wheat rust information exposure  *(no. of times)* |
| Stem rust |  |  |
| Spore deposition 1-53 days after planting *(ln)* | 0.06 (0.04) | 0.15^***^ (0.05) |
| Environmental suitability 1-53 days after planting (0-1) | 2.26^*^ (1.16) | 3.58^**^ (1.57) |
| Yellow rust |  |  |
| Spore deposition 1-53 days after planting *(ln)* | 0.04 (0.04) | 0.04 (0.06) |
| Environmental suitability 1-53 days after planting (0-1) | 1.83^**^ (0.93) | 3.07^**^ (1.24) |
| Age of the household head (years) | -0.01 (0.00) | -0.01 (0.01) |
|  |  |  |
| Years of schooling of the household head | 0.02 (0.01) | 0.02 (0.02) |
| Family members engaging in agriculture (no.) | 0.01 (0.04) | 0.04 (0.06) |
| Household is member in an agricultural institution (yes=1) | 0.13 (0.14) | 0.21 (0.23) |
| Total wheat area cultivated in 2022/23 Meher season (ln ha) | -0.09 (0.09) | -0.06 (0.17) |
| No. years the household has received information on wheat rust from 2015-20 | 0.00 (0.02) | -0.01 (0.04) |
| Constant | -2.59^**^ (1.24) | -3.69^**^ (1.76) |
| var(e.frequency of information exposure) |  | 4.10^***^ (0.84) |
| Observations | 402 | 402 |
| Pseudo *R*^2^ | 0.082 | 0.023 |
| ll | -218.32 | -714.92 |
| chi2 | 133.58 |  |
| F |  | 71.15 |

*Notes: Estimates are marginal effects, showing how the probability of the outcome changes with a one-unit change in the independent variable. Model 1 is a Probit model, with the dependent variable indicating whether the farmer received wheat rust information in the 2022/23 season (1=yes, 0=otherwise). Model 2 is a Tobit model, with the dependent variable representing the frequency of exposure to wheat rust information (right-censored: 0, 1, …, n). Predicted rust spore deposition and environmental suitability are averaged over the first 53 days after planting. Standard errors (in parentheses) are robust and clustered at the district level. *, **, *** denote significance at the 10%, 5%, and 1% levels, respectively. The number of observations is less than 520 due to missing data for 118 observations, i.e., for observations that have planted wheat before the start of weekly rust pressure predictions, or observations with missing household or farm level data.*

**Table S2**. Sampling frame, coverage and response rate by region.

| Region | Sample of Mottaleb, et al. ^24^  (no. obs.) | With phone numbers  (no. obs.) | Coverage rate^#^ | Contacted successfully  (no. obs.) | Response rate^##^ |  |
| --- | --- | --- | --- | --- | --- | --- |
| Amhara | 567 | 411 | 72% | 312 | 76% |  |
| Oromia | 500 | 313 | 63% | 208 | 66% |  |
| Total | 1,067 | 724 | 68% | 526 | 73% | |

*Notes: #Coverage rate= no. observations with phone number/ all observations. ## Response rate = no. observation contacted successfully/ no. observations with phone number.*

Figure S1. Locations of sampled households in Ethiopia. The map was created by the authors using QGIS version 3.34.15 “Prizren” (QGIS Development Team; <https://qgis.org/>)
